# Colorectal Cancer Screening and Health-Related Social Needs in a National Sample of US Adults

**DOI:** 10.1001/jamanetworkopen.2026.6000

**Published:** 2026-04-09

**Authors:** Aldenise P. Ewing, Fode Tounkara, Wayne R. Lawrence, Dede K. Teteh-Brooks, Nicolas Nelson, Subhankar Chakraborty, Emre Sezgin, David Haynes, Timiya S. Nolan, Chyke Doubeni

**Affiliations:** 1 Division of Epidemiology, The Ohio State University College of Public Health, Columbus; 2 The Ohio State University College of Medicine, Columbus; 3 Department of Epidemiology and Biostatistics, State University of New York at Albany, Albany; 4 MD Anderson Cancer Center, Houston, Texas; 5 Southeast Healthcare, Columbus, Ohio; 6 Nationwide Children’s Hospital, Columbus, Ohio; 7 Institute for Health Informatics, Department of Medicine, University of Minnesota, Minneapolis; 8 Heersink School of Medicine, University of Alabama Birmingham, Birmingham

## Abstract

**Importance:**

Health-related social needs (HRSNs) may impede colorectal cancer (CRC) screening among US adults, yet population-based evidence on these associations remains limited.

**Objective:**

To examine associations between HRSNs and CRC screening uptake overall and by age group among US adults.

**Design, Setting, and Participants:**

This cross-sectional study used data from the 2023 National Health Interview Survey. Participants were US civilian, noninstitutionalized adults aged 45 to 75 years eligible for CRC screening. Age-stratified analyses were conducted for adults aged 45 to 49 years, 50 to 64 years, and 65 to 75 years. Data analysis was performed from April 2025 to February 2026.

**Exposures:**

Self-reported HRSNs, including housing instability, food insecurity, transportation barriers, and number of unmet needs.

**Main Outcomes and Measures:**

The primary outcome was being up to date with CRC screening according to US Preventive Services Task Force recommendations. Survey-weighted logistic regression models estimated adjusted odds ratios (aORs) and 95% CIs overall and by age group.

**Results:**

The analytic sample included 14 528 adults aged 45 to 75 years, and most participants were aged 50 to 64 years (6940 individuals [52.42%]), female (7788 individuals [51.36%]), insured (13 750 participants [94.07%]), and reported no unmet HRSNs (12 370 participants [85.40%]). Overall, 2158 adults (14.60%) reported at least 1 unmet HRSN. The proportion up to date with CRC screening was 63.91% (9758 adults) overall and increased with age, from 31.01% (586 adults) among adults aged 45 to 49 years to 64.24% (4539 adults) among those aged 50 to 64 years, and 80.85% (4633 adults) among those aged 65 to 75 years. In adjusted models, housing instability (aOR, 0.82; 95% CI, 0.67-0.99) and transportation barriers (aOR, 0.78; 95% CI, 0.64-0.95) were associated with lower odds of being up to date with CRC screening. Screening odds declined with increasing number of unmet HRSNs, including among adults reporting 1 unmet need (aOR, 0.84; 95% CI, 0.72-0.98). Associations were most pronounced among adults aged 50 to 64 years, among whom housing instability (aOR, 0.77; 95% CI, 0.61-0.97), transportation barriers (aOR, 0.71; 95% CI, 0.56-0.91), and reporting 1 HRSN (aOR, 0.80; 95% CI, 0.66-0.97) were associated with lower odds of being up to date with screening.

**Conclusions and Relevance:**

In this cross-sectional study, unmet HRSNs were associated with lower CRC screening uptake, particularly among adults aged 50 to 64 years. Addressing HRSNs may support age-specific strategies to improve CRC screening.

## Key Points

**Question:**

Is colorectal cancer screening uptake associated with health-related social needs among US adults, and do these associations differ by age group?

**Findings:**

In this cross-sectional study of 14 528 US adults eligible for colorectal cancer screening, unmet health-related social needs were associated with lower likelihood of being up to date with screening. The magnitude and direction of these associations varied across age groups.

**Meaning:**

These findings suggest that addressing health-related social needs may be important for improving colorectal cancer screening uptake, particularly when considering age-specific differences.

## Teaser

This cross-sectional study examines associations between health-related social needs and colorectal cancer screening uptake overall and by age group among US adults.

## Introduction

Colorectal cancer (CRC) is the second leading cause of cancer-related deaths in the US, despite being largely preventable through routine screening.^1,2^ The US Preventive Services Task Force (USPSTF) recommends that adults at average risk and aged 45 to 75 years undergo regular CRC screening via stool-based or direct visualization tests at specified intervals.^3^ Early detection through screening significantly improves treatment outcomes and survival, yet national screening rates remain suboptimal,^4^ particularly among younger adults, those facing socioeconomic disadvantages, and those identifying as minoritized racial and ethnic populations.^5^

Health-related social needs (HRSNs)—defined as adverse social conditions that affect health outcomes—have emerged as critical determinants of CRC screening behavior.^6,7,8^ The Centers for Medicare & Medicaid Services (CMS) recently underscored the relevance of these needs, issuing guidance in 2023 to prioritize food insecurity, housing instability, transportation barriers, utility difficulties, and interpersonal safety in federal health care initiatives.^9^ Social needs barriers are associated with delayed preventive care,^10^ including CRC screening.^7,11,12^ These HRSNs represent the lived consequences of unmet social determinants of health and disproportionately affect low-income and racially or ethnically minoritized populations.^13,14^

Although existing literature has linked individual HRSNs, such as financial strain^15^ or transportation challenges, to lower CRC screening rates, less is known how co-occurring HRSNs interact to influence screening behavior. Furthermore, following the 2021 USPSTF recommendation to lower the CRC screening initiation age to 45 years, it is increasingly important for national studies to include younger cohorts to identify and address persistent social barriers throughout the lifespan. Therefore, this study evaluated age-stratified associations between HRSNs and CRC screening among US adults aged 45 to 75 years. We hypothesized that individuals reporting HRSNs would be less likely to have completed CRC screening and that associations would differ by age.

## Methods

### Study Design and Data Source

We conducted a cross-sectional analysis of the 2023 National Health Interview Survey (NHIS), a nationally representative survey of the civilian, noninstitutionalized US population. A description of the NHIS, including its methods, has been published elsewhere.^16^ The NHIS uses a multistage, stratified sampling design to ensure that it accurately represents various demographic groups. Racial and ethnic minoritized populations and older adults were oversampled to improve the precision of subgroup estimates, and NHIS survey weights were applied in all analyses to account for this sampling design and produce nationally representative estimates. Data were collected in 2023 through in-person interviews led by trained interviewers. All analyses incorporated NHIS sampling weights, strata, and primary sampling units to account for the complex survey design and generate nationally representative estimates.

This study used publicly available, deidentified NHIS data and was exempt from institutional review board review; informed consent was waived. This study followed the Strengthening the Reporting of Observational Studies in Epidemiology (STROBE) reporting guidelines.^17^

### Population

The analytic sample included adults aged 45 to 75 years, consistent with USPSTF screening eligibility for average-risk adults.^3^ Individuals with missing data on CRC screening status or HRSNs were excluded. To restrict analyses to routine CRC screening, participants were excluded if their most recent colonoscopy was reported as because of a problem or follow-up to an earlier test or screening examination (vs part of a routine examination) or if they reported a clinician recommendation in the past 12 months to be tested to look for problems in the colon or rectum.

### Outcome Variable

The primary outcome was self-reported CRC screening. Screening was defined according to USPSTF guidelines.^3^

### HRSN Variables

HRSNs were assessed using NHIS items on food insecurity, housing instability, and transportation barriers and coded as binary indicators (yes or no). Housing instability reflected difficulty paying rent, mortgage, or utilities in the past 12 months; food insecurity reflected inadequate household food availability; and transportation barriers reflected difficulty obtaining transportation to medical care. We also created a cumulative HRSN measure (0, 1, or ≥2 unmet needs). This approach enabled us to examine both the individual and additive associations of HRSNs on CRC screening behavior.

### Covariates

Covariates included age group (45-49, 50-64, or 65-75 years), sex, and race and ethnicity (Hispanic, non-Hispanic Black, non-Hispanic White, and non-Hispanic other). The other race category included respondents identifying as American Indian or Alaska Native, Native Hawaiian or Other Pacific Islander, or multiracial. The other ethnicity category included respondents who did not identify as Hispanic or Latino or who reported multiple ethnicities. Race and ethnicity were self-reported by participants in the NHIS using standardized categories defined by the survey and classified by respondents, not investigators. Race and ethnicity were included to account for known disparities in access to preventive care, including CRC screening, and to improve the interpretability and generalizability of population-level disparities. Socioeconomic characteristics included education, income-to-poverty ratio (annual household income of <200%, 200%-399%, or ≥400% of the federal poverty level [FPL]), and marital status. Health care access measures included insurance status and internet access (yes or no). Given the increasing reliance on digital platforms for health-related communication and service delivery, internet access has emerged as a super social determinant of health and health care access in the US; thus, its inclusion in our study allows for a more comprehensive assessment of covariates influencing CRC screening behavior.^18,19^ All covariates were derived from self-reported NHIS responses using standard coding.

### Study Size

The final analytic sample size was determined by the number of NHIS respondents aged 45 to 75 years with complete data on CRC screening and HRSNs. Weighted estimates reflected the US adult population meeting these criteria.

### Statistical Analysis

Data analysis was performed from April 2025 to February 2026. We used weighted descriptives to summarize the characteristics of the study population. We presented categorical variables as weighted frequencies and percentages, and continuous variables as weighted means and SDs.

We used weighted univariable and multivariable logistic regression models to evaluate the associations between individual HRSNs (ie, housing instability, food insecurity, and transportation barriers), cumulative HRSN burden, and CRC screening. Univariable logistic regression analyses were performed for each candidate variable to identify factors associated with CRC screening outcomes. Multivariable models were constructed using forward selection without univariable prescreening. Candidate covariates were entered sequentially on the basis of statistical significance, with inclusion determined using a single criterion (*P* < .05). This approach was chosen to balance model parsimony with stability while accounting for correlated sociodemographic and access-related factors. Adjusted odds ratios (aORs) and 95% CIs were reported for both univariable and multivariable analyses.

Age-stratified analyses were conducted to assess whether associations between HRSNs and CRC screening differed across age groups (45-49, 50-64, and 65-75 years). Separate survey-weighted multivariable logistic regression models were fitted within each age group, applying the same forward selection strategy. Age group was included as a covariate in overall models to account for its association with screening behavior and to facilitate comparison with age-stratified estimates.

As a sensitivity analysis, we examined associations between HRSNs and CRC screening modality among screened respondents, comparing colonoscopy-based screening with stool-based screening methods. These analyses were conducted using survey-weighted descriptive comparisons and regression models restricted to respondents who were up to date with screening.

All statistical tests were 2 sided, with a significance threshold set at *P* < .05. All analyses were performed using R statistical software version 4.3.1 (R Project for Statistical Computing), utilizing the survey package to properly account for the complex sampling design of the NHIS, and to obtain accurate variance estimates.

## Results

### Study Population Characteristics

Table 1 presents the weighted distributions of descriptive characteristics of the study population aged 45 to 75 years (113 688 944 individuals). The analytic sample included 14 528 adults aged 45 to 75 years. Overall, most were aged 50 to 64 years (6940 individuals [52.42%]) and female (7788 individuals [51.36%]). With regard to race and ethnicity, 1741 individuals (14.51%) were Hispanic, 1648 (11.06%) were non-Hispanic Black, 10 198 (66.72%) were non-Hispanic White, and 941 (7.71%) were non-Hispanic other races. Educational attainment of a bachelor’s degree or higher was reported by 5425 respondents (33.80%), whereas 6523 (46.80%) reported a household income-to-poverty ratio of 400% or more of the FPL. Most participants were insured (13 750 participants [94.07%]) and reported having internet access (13 394 participants [93.39%]). Approximately 14.60% of adults (2158 participants) reported at least 1 unmet HRSN. Specifically, 8.21% (1171 participants) experienced food insecurity, 6.54% (902 participants) reported housing instability, and 6.00% (915 participants) encountered transportation barriers; these categories were not mutually exclusive, as individuals could report multiple needs.

**Table 1.** Cohort Characteristics

Characteristic	Participants, No. (%) (N = 14 528)^a^
Colorectal cancer screening status	
Not screened	4770 (36.09)
Screened	9758 (63.91)
Housing instability	
No	13 176 (93.46)
Yes	902 (6.54)
Food insecurity	
No	12 931 (91.79)
Yes	1171 (8.21)
Transportation barrier	
No	13 181 (94.00)
Yes	915 (6.00)
No. of unmet health-related social needs	
0	12 370 (85.40)
1	1493 (10.15)
≥2	665 (4.45)
Age group, y	
45-49	1879 (16.52)
50-64	6940 (52.42)
65-75	5709 (31.06)
Sex	
Female	7788 (51.36)
Male	6740 (48.64)
Race and ethnicity	
Hispanic	1741 (14.51)
Non-Hispanic Black	1648 (11.06)
Non-Hispanic White	10 198 (66.72)
Non-Hispanic other^b^	941 (7.71)
Education	
Less than high school	1276 (11.31)
High school graduate	3680 (26.13)
Some college	2071 (14.54)
Associate degree	2012 (14.21)
Bachelor’s degree or higher	5425 (33.80)
Income-to-poverty ratio, percentage of federal poverty level	
<200	3913 (25.24)
200-399	4092 (27.96)
≥400	6523 (46.80)
Marital status	
Married	7366 (64.46)
Living as unmarried couple	590 (5.40)
Neither	6207 (30.14)
Insurance status	
Not covered	754 (5.93)
Covered	13 750 (94.07)
Internet access	
No	1128 (6.61)
Yes	13 394 (93.39)
Geographic region	
Northeast	2268 (17.73)
Midwest	3232 (20.86)
South	5478 (38.57)
West	3550 (22.83)

^a^ Cells show unweighted counts and weighted column percentages.

^b^ The non-Hispanic other category includes respondents identifying as American Indian or Alaska Native, Native Hawaiian or Other Pacific Islander, Asian, or multiracial.

### CRC Screening Rates by Sociodemographic and Health Access Characteristics

The overall CRC screening percentage among the sample was 63.91% (9758 participants) (Table 1). As shown in Table 2, CRC screening percentages increased across age groups, with 586 adults aged 45 to 49 years (31.01%), 4539 aged 50 to 64 years (64.24%), and 4633 aged 65 to 75 years (80.85%) reporting being screened. CRC screening percentages were higher among participants who were non-Hispanic White (7111 participants [66.94%]), married (5107 participants [66.15%]), attained a bachelor’s degree or higher (3914 participants [69.75%]), earned 400% or more than the FPL (4615 participants [68.32%]), were insured (9546 participants [66.38%]), who had internet access (9083 participants [64.51%]), and resided in the Northeast region of the US (1598 participants [67.68%]) compared with their respective counterparts.

**Table 2.** Sociodemographic and Health Access Characteristics by Colorectal Cancer Screening Status

Characteristic	Participants, No. (%)		*P* value^b^
	Not screened (n = 4770 [36.09%])^a^	Screened (n = 9758 [63.91%])^a^	
No. of unmet health-related social needs			
0	3878 (34.48)	8492 (65.52)	<.001
1	587 (43.83)	906 (56.17)	
≥2	305 (49.35)	360 (50.65)	
Housing instability			
No	4166 (34.84)	9010 (65.16)	<.001
Yes	404 (48.19)	498 (51.81)	
Food insecurity			
No	4085 (34.79)	8846 (65.21)	<.001
Yes	499 (46.70)	672 (53.30)	
Transportation barrier			
No	4209 (35.15)	8972 (64.85)	<.001
Yes	371 (45.26)	544 (54.74)	
Age group, y			
45-49	1293 (68.99)	586 (31.01)	<.001
50-64	2401 (35.76)	4539 (64.24)	
65-75	1076 (19.15)	4633 (80.85)	
Sex			
Female	2502 (35.66)	5286 (64.34)	.33
Male	2268 (36.55)	4472 (63.45)	
Race and ethnicity			
Hispanic	776 (47.97)	965 (52.03)	<.001
Non-Hispanic Black	534 (34.89)	1114 (65.11)	
Non-Hispanic White	3087 (33.06)	7111 (66.94)	
Non-Hispanic other^c^	373 (41.73)	568 (58.27)	
Education			
Less than high school	584 (47.81)	692 (52.19)	<.001
High school graduate	1372 (40.38)	2308 (59.62)	
Some college	650 (34.15)	1421 (65.85)	
Associate degree	622 (34.15)	1390 (65.85)	
Bachelor’s degree or higher	1511 (30.25)	3914 (69.75)	
Income-to-poverty ratio, percentage of federal poverty level			
<200	1499 (43.02)	2414 (56.98)	<.001
200-399	1363 (37.23)	2729 (62.77)	
≥400	1908 (31.68)	4615 (68.32)	
Marital status			
Married	2259 (33.85)	5107 (66.15)	<.001
Living as unmarried couple	242 (44.32)	348 (55.68)	
Neither	2103 (38.39)	4104 (61.61)	
Insurance status			
Not covered	555 (74.63)	199 (25.37)	<.001
Covered	4204 (33.62)	9546 (66.38)	
Internet access			
No	455 (44.55)	673 (55.45)	<.001
Yes	4311 (35.49)	9083 (64.51)	
Geographic region			
Northeast	670 (32.32)	1598 (67.68)	<.001
Midwest	1071 (36.78)	2161 (63.22)	
South	1916 (38.16)	3562 (61.84)	
West	1113 (34.90)	2437 (65.10)	

^a^ Cells show unweighted counts and weighted column percentages.

^b^ *P* values from Rao-Scott χ^2^ tests (categorical) or design-based *t* tests (continuous).

^c^ The non-Hispanic other category includes respondents identifying as American Indian or Alaska Native, Native Hawaiian or Other Pacific Islander, Asian, or multiracial.

### CRC Screening Rates by HRSN and Number of Unmet HRSN Across Age Groups

On the basis of Rao-Scott χ^2^ tests, CRC screening status differed across HRSN categories overall and by age group (Table 3). Overall, screening status differed by housing instability, food insecurity, transportation barriers, and number of unmet HRSNs. For example, among those reporting housing instability, 404 (48.19%) were screened and 498 (51.81%) were not screened, compared with 4166 (34.84%) screened and 9010 (65.16%) not screened among adults without housing instability.

**Table 3.** Colorectal Cancer Screening Rates by HRSN and Number of Unmet HRSNs Across Age Groups

Characteristic	Overall (N = 14 528)			Age 45-49 y (n = 1879)			Age 50-64 y (n = 6940)			Age 65-75 y (n = 5709)		
	Participants, No. (%)^a^		*P* value^b^	Participants, No. (%)^a^		*P* value^b^	Participants, No. (%)^a^		*P* value^b^	Participants, No. (%)^a^		*P* value^b^
	Screened	Not screened		Screened	Not screened		Screened	Not screened		Screened	Not screened	
Housing instability												
No	4166 (34.84)	9010 (65.16)	<.001	1118 (68.34)	531 (31.66)	.39	2076 (34.54)	4129 (65.46)	<.001	972 (18.57)	4350 (81.43)	.01
Yes	404 (48.19)	498 (51.81)		119 (72.41)	39 (27.59)		224 (46.51)	280 (53.49)		61 (26.35)	179 (73.65)	
Food insecurity												
No	4085 (34.79)	8846 (65.21)	<.001	1104 (68.09)	528 (31.91)	.15	2032 (34.49)	4055 (65.51)	<.001	949 (18.54)	4263 (81.51)	.004
Yes	499 (46.70)	672 (53.30)		135 (74.62)	42 (25.38)		277 (45.85)	361 (54.15)		87 (25.69)	269 (74.31)	
Transportation barrier												
No	4209 (35.15)	8972 (64.85)	<.001	1151 (68.56)	539 (31.44)	.64	2098 (34.71)	4156 (65.29)	<.001	960 (18.54)	4277 (81.46)	.009
Yes	371 (45.26)	544 (54.74)		87 (71.02)	31 (28.98)		208 (47.18)	259 (52.82)		76 (25.78)	254 (74.22)	
No. of HRSNs												
0	3878 (34.48)	8492 (65.52)	<.001	1054 (68.02)	507 (31.98)	.27	1905 (33.96)	3891 (66.04)	<.001	919 (18.48)	4094 (81.52)	.002
1	587 (43.83)	906 (56.17)		152 (74.60)	54 (25.40)		330 (44.26)	449 (55.74)		105 (22.23)	403 (77.77)	
≥2	305 (49.35)	360 (50.65)		87 (72.61)	25 (27.39)		166 (47.86)	199 (52.14)		52 (29.77)	136 (70.23)	

Abbreviation: HRSN, health-related social needs.

^a^ Cells show unweighted counts (weighted row percentages) based on survey design.

^b^ *P* values are based on *t* test for continuous variables; Rao-Scott χ^2^ tests for categorical variables.

Age-stratified analyses showed that differences were most pronounced among adults aged 50 to 64 years. In this age group, among adults reporting housing instability, 224 (46.51%) were screened and 280 (53.49%) were not screened, compared with 2076 (34.54%) screened and 4129 (65.46%) not screened among adults without housing instability. Among adults aged 65 to 75 years, screening status also differed significantly across HRSN categories and by number of unmet needs; for example, 61 adults (26.35%) reporting housing instability were screened and 179 (73.65%) were not screened, compared with 972 (18.57%) screened and 4350 (81.43%) not screened among adults without housing instability. On the basis of the number of unmet needs within this age group, the proportion screened was 18.48% (919 of 4971 participants) among those with no unmet needs, 22.23% (105 of 508 participants) among those with 1 unmet need, and 29.77% (52 of 188 participants) among those with 2 or more unmet needs. No statistically significant differences were observed among adults aged 45 to 49 years. These descriptive comparisons reflect unadjusted row percentages in Table 3 and do not account for differences in age or other sociodemographic characteristics.

### Associations Between HRSNs, Number of Unmet HRSNs, and CRC Screening Across Age Groups

In multivariable survey-weighted logistic regression models, selected HRSNs were associated with lower odds of being up to date with CRC screening, with variation by age group (Table 4 and eTables 1-3 in Supplement 1). Overall, housing instability (aOR, 0.82; 95% CI, 0.67-0.99; *P* = .04) and transportation barriers (aOR, 0.78; 95% CI, 0.64-0.95; *P* = .01) were associated with lower odds of being up to date with screening, whereas food insecurity was not.

**Table 4.** Associations Between HRSNs and Being Up to Date with Colorectal Cancer Screening, Overall and by Age Group

Characteristic	Overall (N = 14 528)		Age 45-49 y (n = 1879)		Age 50-64 y (n = 6940)		Age 65-75 y (n = 5709)	
	aOR (95% CI)^a^	*P* value	aOR (95% CI)^a^	*P* value	aOR (95% CI)^a^	*P* value	aOR (95% CI)^a^	*P* value
Housing instability (reference, no)	0.82 (0.67-0.99)	.04	0.94 (0.58-1.54)	.81	0.77 (0.61-0.97)	.03	0.86 (0.60-1.24)	.43
Food insecurity (reference, no)	0.92 (0.77-1.09)	.32	0.99 (0.62-1.59)	.97	0.90 (0.72-1.12)	.34	0.90 (0.66-1.23)	.50
Transportation barrier (reference, no)	0.78 (0.64-0.95)	.01	0.98 (0.59-1.65)	.95	0.71 (0.56-0.91)	.007	0.83 (0.60-1.16)	.28
No. of unmet HRSNs (reference, 0)								
1	0.84 (0.72-0.98)	.03	0.85 (0.53-1.35)	.49	0.80 (0.66-0.97)	.02	0.99 (0.74-1.32)	.94
≥2	0.81 (0.65-1.02)	.07	1.00 (0.54-1.85)	.99	0.77 (0.57-1.02)	.07	0.77 (0.52-1.14)	.20

Abbreviations: aOR, adjusted odds ratio; HRSN, health-related social need.

^a^ aORs with 95% CI were calculated from multivariable survey-weighted logistic regression. Adjusted covariates for overall aOR included age group, sex, race and ethnicity, education, federal poverty level, marital status, insurance status, internet access, and region. Adjusted covariates for age group aOR included sex, race and ethnicity, education, federal poverty level, marital status, insurance status, internet access, and region.

Age-stratified analyses indicated that these associations were most pronounced among adults aged 50 to 64 years. In this age group, housing instability (aOR, 0.77; 95% CI, 0.61-0.97; *P* = .03) and transportation barriers (aOR, 0.71; 95% CI, 0.56-0.91; *P* = .007) were associated with lower odds of being up to date with screening. In addition, reporting 1 unmet HRSN was associated with lower odds of up-to-date screening status, both overall (aOR, 0.84; 95% CI, 0.72-0.98; *P* = .03) and among adults aged 50 to 64 years (aOR, 0.80; 95% CI, 0.66-0.97; *P* = .02), whereas reporting 2 or more unmet HRSNs was not statistically significant. Among adults aged 45 to 49 years and 65 to 75 years, no individual HRSN or number of unmet HRSNs was significantly associated with being up to date with CRC screening after adjustment.

### Sensitivity Analyses

Exploratory sensitivity analyses are presented in eTable 4 in Supplement 1. Associations between HRSNs and CRC screening were generally consistent with the primary findings across alternative model specifications. When screening modality was examined among screened respondents, unmet social needs were more often associated with lower uptake of colonoscopy-only screening than stool-based screening. Inclusion of geographic region and age group did not materially change effect estimates.

## Discussion

In this nationally representative cross-sectional sample of US adults aged 45 to 75 years, we found that unmet HRSNs were associated with lower likelihood of being up to date with CRC screening, with notable variation by age group. Descriptive analyses demonstrated statistically significant differences in screening distributions across HRSNs categories overall and among adults aged 50 to 64 and 65 to 75 years. However, after accounting for sociodemographic and health access factors in multivariable models, only housing instability and transportation barriers remained independently associated with screening, and these associations were most pronounced among adults aged 50 to 64 years, a population long included in CRC screening guidelines.^20^

The contrast between unadjusted and adjusted findings highlights the importance of distinguishing descriptive screening distributions from independent associations. In unadjusted analyses, adults reporting 1 or more unmet social need appeared more likely to be screened within certain age strata, particularly among adults aged 65 to 75 years. These patterns likely reflect confounding by age, insurance coverage, health care utilization, or greater contact with clinical settings where screening opportunities occur. After adjustment for these factors, no individual HRSN or cumulative burden remained significantly associated with screening among adults aged 65 to 75 years, suggesting that observed descriptive differences in this age group were largely explained by underlying population characteristics rather than social needs alone.

In contrast, among adults aged 50 to 64 years, who are typically not yet Medicare eligible and may experience greater variability in insurance coverage and health care access, housing instability and transportation barriers were consistently associated with lower odds of screening, even after adjustment. This finding suggests that structural barriers may play a particularly key role in shaping preventive care engagement during midlife, when competing financial, caregiving, and occupational demands are common.^21^

HRSNs are critical, modifiable barriers that influence individuals’ ability to access and engage in preventive care, including CRC screening. In 2023, a reported 13.5% of US households were food insecure, without access at all times to enough food for an active, healthy life for all household members.^3,22^ Food insecurity is associated with chronic illnesses and lower self-reported health status among older adults.^23^ Housing instability has affected over 42 million US households, with renters from lower-income and historically underserved groups facing the greatest burden.^24^ Owing to long-standing differences in housing opportunities, these populations are more likely to live in poor-quality housing and disadvantaged neighborhoods, which contributes to ongoing differences in health outcomes.^13,25,26^ Transportation barriers, which affect 5.7% of US individuals, are especially prevalent among women, younger adults, and those with lower socioeconomic status.^27^

Our findings reinforce and extend prior work demonstrating that social adversity undermines engagement in preventive health services.^28,29,30^ Previous studies have shown that individual HRSNs, particularly financial strain and transportation challenges, are linked to lower cancer screening uptake.^7^ More recent studies have also linked severe housing cost burden and premature cancer mortality,^31^ underscoring the broader health consequences of unmet social needs. However, few have examined how co-occurring HRSNs influence screening behavior across age groups. In our study, housing instability and transportation barriers were factors significantly associated with reduced CRC screening, particularly among those aged 50 to 64 years. These results suggest that social context, especially the accumulation of multiple unmet needs, remains a critical barrier in preventive care, even in populations with long-standing screening recommendations.

Adults aged 50 to 64 years may face distinct barriers because of overlapping caregiving roles, lower access to employer-sponsored insurance, or difficulties navigating screening pathways. Although individuals in this group have been age-eligible for screening for decades, our results indicate that guideline awareness alone is insufficient to overcome structural challenges. In contrast, associations among adults aged 45 to 49 years and 65 to 75 years were attenuated, suggesting that younger adults may not yet be fully engaged with preventive care, and older adults may benefit from greater insurance coverage (eg, Medicare).

From a policy perspective, these findings support ongoing efforts to integrate social needs screening into routine preventive care, as recommended by CMS^32^ and other national organizations. Targeted interventions, such as community health workers, digital navigators, or cross-sector partnerships, could help identify and mitigate barriers at key prevention windows, particularly for individuals reporting multiple unmet needs.

### Limitations

This study has several limitations. First, all variables, including CRC screening and HRSNs, were based on self-report and may be subject to recall or social desirability bias; however, self-reported screening data from the NHIS have demonstrated acceptable validity in prior studies.^33^ Second, because of the cross-sectional design, causal inferences cannot be made, and we cannot determine whether unmet HRSNs preceded or followed CRC screening behavior. Third, although we adjusted for multiple sociodemographic characteristics, unmeasured confounding may remain. For example, we did not include measures of health literacy, practitioner communication quality, or social support, which may influence both HRSNs and CRC screening uptake. In addition, some HRSN domains prioritized by CMS (eg, interpersonal safety or utility assistance) were not captured in the NHIS and, thus, were not included in our cumulative assessment.

Fourth, despite applying multiple criteria to restrict analyses to routine CRC screening, the limited availability of survey items to definitively distinguish screening among average-risk adults from diagnostic or surveillance testing may have resulted in some outcome misclassification. Fifth, although age-stratified analyses revealed meaningful differences across subgroups, including adults newly eligible for screening, the relatively small proportion of respondents with multiple HRSNs limited the precision of estimates in some strata. Nevertheless, our findings provide robust evidence from a nationally representative sample and add to the growing literature on social determinants in preventive cancer care.

## Conclusions

Unmet HRSNs, especially housing instability and transportation barriers, are associated with lower odds of CRC screening, with the greatest impact observed among adults aged 50 to 64 years. Our findings underscore the importance of addressing cumulative social risk within both clinical and community-based prevention efforts. Interventions that integrate social care into health care workflows and address multiple barriers simultaneously may help reduce persistent disparities in CRC screening and outcomes.

## Notes

Supplement 1. eTable 1. Unadjusted and Adjusted Associations Between Housing Instability and Colorectal Cancer Screening Among U.S. Adults Aged 45–75 Years eTable 2. Unadjusted and Adjusted Associations Between Food Insecurity and Colorectal Cancer Screening Among U.S. Adults Aged 45–75 Years eTable 3. Unadjusted and Adjusted Associations Between Transportation Barrier and Colorectal Cancer Screening Among U.S. Adults Aged 45–75 Years eTable 4. Distribution of Colorectal Cancer Screening Modality by Health-Related Social Needs

Supplement 2. Data Sharing Statement
