# Supplementary material for: Colorectal Cancer Screening and Health-Related Social Needs in a National Sample of US Adults
Source: JAMA Netw Open. 2026 Apr 9;9(4):e266000. doi: 10.1001/jamanetworkopen.2026.6000 (PMC13067003; doi:10.1001/jamanetworkopen.2026.6000)
Supplement: Supplement 1. — eTable 1. Unadjusted and Adjusted Associations Between Housing Instability and Colorectal Cancer Screening Among U.S. Adults Aged 45–75 Years eTable 2. Unadjusted and Adjusted Associations Between Food Insecurity and Colorectal Cancer Screening Among U.S. Adults Aged 45–75 Years eTable 3. Unadjusted and Adjusted Associations Between Transportation Barrier and Colorectal Cancer Screening Among U.S. Adults Aged 45–75 Years eTable 4. Distribution of Colorectal Cancer Screening Modality by Health-Related Social Needs [file jamanetwopen-e266000-s001.pdf]

Supplemental Online Content

Ewing AP, Tounkara F, Lawrence WR, et al. Colorectal cancer screening and health-related social needs in a national sample of US adults. *JAMA Netw Open*. 2026;9(4):e266000. doi:10.1001/jamanetworkopen.2026.6000

eTable 1. Unadjusted and Adjusted Associations Between Housing Instability and Colorectal Cancer Screening Among U.S. Adults Aged 45–75 Years
eTable 2. Unadjusted and Adjusted Associations Between Food Insecurity and Colorectal Cancer Screening Among U.S. Adults Aged 45–75 Years
eTable 3. Unadjusted and Adjusted Associations Between Transportation Barrier and Colorectal Cancer Screening Among U.S. Adults Aged 45–75 Years
eTable 4. Distribution of Colorectal Cancer Screening Modality by Health-Related Social Needs

This supplemental material has been provided by the authors to give readers additional information about their work.

© 2026 Ewing AP et al. *JAMA Network Open*.

eTable 1. Unadjusted and Adjusted Associations Between Housing Instability and Colorectal Cancer Screening Among U.S. Adults Aged 45–75 Years

	Univariate Analysis			Multivariate Analysis		
**Characteristic**[1]	**OR**	**95% CI**	**p-value**	**OR**	**95% CI**	**p-value**[2]
**Housing instability**						
No	—	—		—	—	
Yes	0.57	0.49, 0.67	**<0.001**	0.82	0.67, 0.99	**0.039**
**Age group (years)**						
45-49	—	—		—	—	
50-64	4.00	3.51, 4.55	**<0.001**	4.26	3.71, 4.88	**<0.001**
65-75	9.39	8.21, 10.7	**<0.001**	9.69	8.39, 11.2	**<0.001**
**Gender**						
Male	—	—		—	—	
Female	1.04	0.96, 1.12	0.3	1.01	0.93, 1.10	0.8
**Race/Ethnicity**						
Hispanic	—	—		—	—	
Non-Hispanic Black	1.72	1.44, 2.05	**<0.001**	1.49	1.21, 1.85	**<0.001**
Non-Hispanic Other	1.29	1.06, 1.57	**0.012**	0.85	0.68, 1.05	0.13
Non-Hispanic White	1.87	1.63, 2.14	**<0.001**	1.15	0.98, 1.34	0.087
**Education**						
Less than HS	—	—		—	—	
HS Graduate	1.35	1.15, 1.59	**<0.001**	1.08	0.90, 1.30	0.4
Some College	1.77	1.49, 2.09	**<0.001**	1.31	1.08, 1.59	**0.007**
Associate Degree	1.77	1.47, 2.12	**<0.001**	1.27	1.02, 1.57	**0.033**
Bachelor+	2.11	1.81, 2.46	**<0.001**	1.60	1.32, 1.95	**<0.001**
**Income-to-poverty ratio**						
<200% FPL	—	—		—	—	
200%-399% FPL	1.27	1.14, 1.42	**<0.001**	1.01	0.89, 1.15	0.8
>=400% FPL	1.63	1.47, 1.80	**<0.001**	1.21	1.05, 1.39	**0.009**

© 2026 Ewing AP et al. *JAMA Network Open*.

	Univariate Analysis			Multivariate Analysis		
**Characteristic**[1]	**OR**	**95% CI**	**p-value**	**OR**	**95% CI**	**p-value**[2]
**Marital status**						
Married	—	—		—	—	
Living as unmarried couple	0.64	0.54, 0.77	**<0.001**	0.90	0.73, 1.10	0.3
Neither	0.82	0.76, 0.89	**<0.001**	0.83	0.75, 0.91	**<0.001**
**Insurance status**						
Not covered	—	—		—	—	
Covered	5.81	4.71, 7.16	**<0.001**	3.42	2.72, 4.30	**<0.001**
**Internet access**						
No	—	—		—	—	
Yes	1.46	1.27, 1.69	**<0.001**	1.40	1.18, 1.66	**<0.001**
**Geographic region**						
Northeast	—	—		—	—	
Midwest	0.82	0.71, 0.95	**0.008**	0.80	0.68, 0.94	**0.006**
South	0.77	0.68, 0.88	**<0.001**	0.81	0.71, 0.93	**0.004**
West	0.89	0.77, 1.03	0.11	1.01	0.86, 1.18	>0.9

[1]The non-Hispanic Other category includes respondents identifying as American Indian or Alaska Native,
Native Hawaiian or Other Pacific Islander, Asian, or multiracial.

[2]Adjusted odd ratio (aOR) from multivariate logistic regression. Adjusted covariables were selected through a stepwise (forward selection) approach.
All covariates were selected.
P-values are from Wald tests. Bold indicates p < 0.05.

Abbreviations: CI = Confidence Interval, OR = Odds Ratio

© 2026 Ewing AP et al. *JAMA Network Open*.

eTable 2. Unadjusted and Adjusted Associations Between Food Insecurity and Colorectal Cancer Screening Among U.S. Adults Aged 45–75 Years

	Univariate Analysis			Multivariate Analysis		
**Characteristic**[1]	**OR**	**95% CI**	**p-value**	**OR**	**95% CI**	**p-value**[2]
**Food insecurity**						
No	—	—		—	—	
Yes	0.61	0.53, 0.70	**<0.001**	0.92	0.77, 1.09	0.3
**Age group (years)**						
45-49	—	—		—	—	
50-64	4.00	3.51, 4.55	**<0.001**	4.25	3.71, 4.87	**<0.001**
65-75	9.39	8.21, 10.7	**<0.001**	9.73	8.42, 11.2	**<0.001**
**Gender**						
Male	—	—		—	—	
Female	1.04	0.96, 1.12	0.3	1.01	0.93, 1.10	0.8
**Race/Ethnicity**						
Hispanic	—	—		—	—	
Non-Hispanic Black	1.72	1.44, 2.05	**<0.001**	1.48	1.20, 1.82	**<0.001**
Non-Hispanic Other	1.29	1.06, 1.57	**0.012**	0.85	0.68, 1.05	0.13
Non-Hispanic White	1.87	1.63, 2.14	**<0.001**	1.14	0.97, 1.33	0.11
**Education**						
Less than HS	—	—		—	—	
HS Graduate	1.35	1.15, 1.59	**<0.001**	1.09	0.90, 1.32	0.4
Some College	1.77	1.49, 2.09	**<0.001**	1.32	1.08, 1.60	**0.006**
Associate Degree	1.77	1.47, 2.12	**<0.001**	1.27	1.02, 1.58	**0.030**
Bachelor+	2.11	1.81, 2.46	**<0.001**	1.61	1.32, 1.96	**<0.001**
**Income-to-poverty ratio**						
<200% FPL	—	—		—	—	
200%-399% FPL	1.27	1.14, 1.42	**<0.001**	1.02	0.89, 1.16	0.8
>=400% FPL	1.63	1.47, 1.80	**<0.001**	1.23	1.06, 1.42	**0.005**

© 2026 Ewing AP et al. *JAMA Network Open*.

	Univariate Analysis			Multivariate Analysis		
**Characteristic**[1]	**OR**	**95% CI**	**p-value**	**OR**	**95% CI**	**p-value**[2]
**Marital status**						
Married	—	—		—	—	
Living as unmarried couple	0.64	0.54, 0.77	**<0.001**	0.89	0.73, 1.09	0.3
Neither	0.82	0.76, 0.89	**<0.001**	0.83	0.75, 0.91	**<0.001**
**Insurance status**						
Not covered	—	—		—	—	
Covered	5.81	4.71, 7.16	**<0.001**	3.45	2.74, 4.34	**<0.001**
**Internet access**						
No	—	—		—	—	
Yes	1.46	1.27, 1.69	**<0.001**	1.40	1.18, 1.66	**<0.001**
**Geographic region**						
Northeast	—	—		—	—	
Midwest	0.82	0.71, 0.95	**0.008**	0.80	0.68, 0.94	**0.006**
South	0.77	0.68, 0.88	**<0.001**	0.82	0.71, 0.94	**0.005**
West	0.89	0.77, 1.03	0.11	1.00	0.86, 1.17	>0.9

[1]The non-Hispanic Other category includes respondents identifying as American Indian or Alaska Native,
Native Hawaiian or Other Pacific Islander, Asian, or multiracial.

[2]Adjusted odd ratio (aOR) from multivariate logistic regression. Adjusted covariables were selected through a stepwise (forward selection) approach.
All covariates were selected.
P-values are from Wald tests. Bold indicates p < 0.05.

Abbreviations: CI = Confidence Interval, OR = Odds Ratio

© 2026 Ewing AP et al. *JAMA Network Open*.

eTable 3. Unadjusted and Adjusted Associations Between Transportation Barrier and Colorectal Cancer Screening Among U.S. Adults Aged 45–75 Years

	Univariate Analysis			Multivariate Analysis		
**Characteristic**[1]	**OR**	**95% CI**	**p-value**	**OR**	**95% CI**	**p-value**[2]
**Transportation barrier**						
No	—	—		—	—	
Yes	0.66	0.55, 0.78	**<0.001**	0.78	0.64, 0.95	**0.012**
**Age group (years)**						
45-49	—	—		—	—	
50-64	4.00	3.51, 4.55	**<0.001**	4.26	3.72, 4.89	**<0.001**
65-75	9.39	8.21, 10.7	**<0.001**	9.75	8.43, 11.3	**<0.001**
**Gender**						
Male	—	—		—	—	
Female	1.04	0.96, 1.12	0.3	1.01	0.92, 1.10	0.8
**Race/Ethnicity**						
Hispanic	—	—		—	—	
Non-Hispanic Black	1.72	1.44, 2.05	**<0.001**	1.48	1.20, 1.82	**<0.001**
Non-Hispanic Other	1.29	1.06, 1.57	**0.012**	0.86	0.69, 1.06	0.2
Non-Hispanic White	1.87	1.63, 2.14	**<0.001**	1.15	0.98, 1.34	0.079
**Education**						
Less than HS	—	—		—	—	
HS Graduate	1.35	1.15, 1.59	**<0.001**	1.08	0.89, 1.30	0.4
Some College	1.77	1.49, 2.09	**<0.001**	1.32	1.08, 1.60	**0.006**
Associate Degree	1.77	1.47, 2.12	**<0.001**	1.27	1.02, 1.58	**0.031**
Bachelor+	2.11	1.81, 2.46	**<0.001**	1.61	1.32, 1.96	**<0.001**
**Income-to-poverty ratio**						
<200% FPL	—	—		—	—	
200%-399% FPL	1.27	1.14, 1.42	**<0.001**	1.01	0.89, 1.15	0.9
>=400% FPL	1.63	1.47, 1.80	**<0.001**	1.22	1.06, 1.40	**0.006**

© 2026 Ewing AP et al. *JAMA Network Open*.

	Univariate Analysis			Multivariate Analysis		
**Characteristic**[1]	**OR**	**95% CI**	**p-value**	**OR**	**95% CI**	**p-value**[2]
**Marital status**						
Married	—	—		—	—	
Living as unmarried couple	0.64	0.54, 0.77	**<0.001**	0.89	0.73, 1.09	0.3
Neither	0.82	0.76, 0.89	**<0.001**	0.83	0.76, 0.92	**<0.001**
**Insurance status**						
Not covered	—	—		—	—	
Covered	5.81	4.71, 7.16	**<0.001**	3.45	2.74, 4.33	**<0.001**
**Internet access**						
No	—	—		—	—	
Yes	1.46	1.27, 1.69	**<0.001**	1.40	1.18, 1.66	**<0.001**
**Geographic region**						
Northeast	—	—		—	—	
Midwest	0.82	0.71, 0.95	**0.008**	0.79	0.68, 0.93	**0.005**
South	0.77	0.68, 0.88	**<0.001**	0.82	0.71, 0.94	**0.005**
West	0.89	0.77, 1.03	0.11	1.00	0.86, 1.17	>0.9

[1]The non-Hispanic Other category includes respondents identifying as American Indian or Alaska Native,
Native Hawaiian or Other Pacific Islander, Asian, or multiracial.

[2]Adjusted odd ratio (aOR) from multivariate logistic regression. Adjusted covariables were selected through a stepwise (forward selection) approach.
All covariates were selected.
P-values are from Wald tests. Bold indicates $p < 0.05$.

Abbreviations: CI = Confidence Interval, OR = Odds Ratio

© 2026 Ewing AP et al. *JAMA Network Open*.

eTable 4. Distribution of Colorectal Cancer Screening Modality by Health-Related Social Needs

Characteristic	Colonoscopy only n (%) = 6010 (77.44)[1]	Stool-based only n (%) = 1704 (22.56)[1]	p-value[2]
**Housing instability**			<0.001
No	5,601 (95.80%)	1,551 (92.82%)	
Yes	242 (4.20%)	117 (7.18%)	
**Food insecurity**			<0.001
No	5,534 (95.07%)	1,493 (89.44%)	
Yes	314 (4.93%)	175 (10.56%)	
**Transportation barrier**			0.10
No	5,561 (95.56%)	1,559 (94.43%)	
Yes	287 (4.44%)	109 (5.57%)	
**No. of unmet HRSNs**			<0.001
0	5,355 (89.57%)	1,420 (84.15%)	
1	495 (8.01%)	193 (10.14%)	
2+	160 (2.42%)	91 (5.71%)	

[1]Cells show unweighted counts and weighted column percentages accounting for NHIS complex survey design.

[2]P values from Rao–Scott χ^2 tests.

© 2026 Ewing AP et al. *JAMA Network Open*.
