# Supplementary material for: Colorectal Cancer Screening and Health-Related Social Needs in a National Sample of US Adults
Source: JAMA Netw Open. 2026 Apr 9;9(4):e266000. doi: 10.1001/jamanetworkopen.2026.6000 (PMC13067003; doi:10.1001/jamanetworkopen.2026.6000)
Supplement: Supplement 2. — Data Sharing Statement [file jamanetwopen-e266000-s002.pdf]

Data Sharing Statement

Ewing. Colorectal Cancer Screening and Health-Related Social Needs in a National Sample of US Adults. *JAMA Netw Open*. Published April 09, 2026.
doi:10.1001/jamanetworkopen.2026.6000

Data
Data available: No

Additional Information
Explanation for why data not available: This study utilized publicly available data from the 2023 National Health Interview Survey (NHIS), conducted by the National Center for Health Statistics (NCHS) at the Centers for Disease Control and Prevention. The authors acknowledge the NCHS and survey participants for providing access to these nationally representative data. The NHIS dataset is available for public use and can be accessed through the NCHS website: https://www.cdc.gov/nchs/nhis/data-questionnaires-documentation.htm.
